# NeonatAIlogy: The GIRISH (Goal, Input, Role, Iterative Refinement, Safety Verification, and Human Accountability) Framework for Structured, Human-Accountable Artificial Intelligence in Neonatal Intensive Care

**DOI:** 10.7759/cureus.114149

**Published:** 2026-08-07

**Authors:** Girish Gupta, Shantanu Shubham, Richa Joshi, Syed Moiz Ahmed, Naini Puri

**Affiliations:** 1 Neonatology, Graphic Era Deemed to be University, Dehradun, IND; 2 Pediatrics, Graphic Era Deemed to be University, Dehradun, IND

**Keywords:** artificial intelligence, clinical ai governance, girish framework, human accountability, large language models, machine learning, neonatology, nicu, structured clinical ai interaction

## Abstract

Artificial intelligence (AI) chatbots are becoming increasingly accessible at the point of care, including in the neonatal intensive care unit (NICU), yet structured guidance for their safe use remains limited. This technical report reviews AI applications across neonatology and introduces GIRISH (Goal, Input, Role, Iterative refinement, Safety verification, and Human accountability), a practical six-step framework for safe, effective, and human-accountable AI use at the NICU bedside, together with ready-to-adapt prompt templates for eight clinical domains. This is a narrative technical report and conceptual synthesis, not a systematic or scoping review; therefore, it provides an illustrative rather than exhaustive overview of AI applications, and no formal PRISMA-ScR record accounting was undertaken. The report is informed by a narrative literature search of PubMed/MEDLINE, Embase, Scopus, and the Cochrane Library (January 2020 to January 2026) *covering *eight neonatal domains, with the GIRISH framework developed through primary conceptual synthesis. This report maps AI applications in neonatology across five tiers, ranging from rule-based clinical decision support to multimodal agentic systems. GIRISH offers a bedside-ready interaction framework with domain-specific prompt templates, a three-tier safety hierarchy, and a minimum viable adaptation (MV-GIRISH) for resource-limited settings. GIRISH structures how AI is consulted, interrogated, and documented at the bedside. Finally, we propose *NeonatAIlogy* as a conceptual direction - not an already-established subdiscipline - to organize the governance, education, and research infrastructure required for its responsible integration.

## Introduction

Artificial intelligence (AI) is rapidly transforming healthcare by enabling advanced data analysis, predictive modeling, clinical decision support, and natural language interaction across medical specialties. Advances in machine learning, deep learning, foundation models, and large language models (LLMs) have expanded AI from predictive analytics to clinical documentation, evidence synthesis, decision support, and patient communication [1].

For clarity, we distinguish three categories used throughout this report: predictive machine-learning (ML) models, which output a risk score or classification; foundation models and LLMs, which generate free text; and agentic systems, which chain model calls to act with limited autonomy. Throughout, "calibration" denotes the agreement between predicted and observed risk and is distinct from discrimination (the area under the receiver operating characteristic curve, AUROC).

The relevance of AI is particularly evident in low- and middle-income countries (LMICs), where shortages of trained healthcare professionals, rising patient volumes, and resource limitations create opportunities for AI-assisted care delivery. Across maternal and neonatal settings, AI is being explored for risk stratification, physiologic monitoring, early identification of clinical deterioration, telehealth support, and decision-making assistance aimed at improving outcomes [2]. These developments are especially relevant to neonatology, where complex decisions often require integration of large volumes of physiological, laboratory, imaging, and developmental data.

In India and comparable settings, where neonatal services frequently face workforce and infrastructure challenges, early experience with generative AI suggests potential to augment - not replace - family-centered communication: for example, ChatGPT has been used to provide patient-specific answers to parents’ questions in pediatric intensive care, with clinician oversight, illustrating how AI may enhance rather than replace human communication [3].

Consider a familiar scenario. At 3 am, a 28-week, 950 g infant on day of life (DOL) 7 is deteriorating: the fraction of inspired oxygen (FiO_2_) has risen from 0.38 to 0.55 over six hours, C-reactive protein (CRP) is 48 mg/L, the white cell count is 18.4 × 10⁹/L with 74% neutrophils, and the temperature is 37.9°C after seven days of ampicillin. A clinician opens a phone and types a vague query such as “late-onset sepsis in premature baby, what antibiotic?” The response arrives in seconds; it is plausible, authoritative, and may be exactly right or completely wrong for this patient. Without a structured approach, the two are difficult to tell apart. This scenario is illustrative but reflects documented practice: surveys and reviews report that neonatologists worldwide already use AI chatbots - ChatGPT, Gemini, Claude, Copilot, and others - for differential diagnoses, drug-dose checks, guideline summaries, parent-counseling drafts, and discharge letters, and AI increasingly influences multiple aspects of neonatal care [4]. A 2025 study confirmed that AI chatbots achieve substantial accuracy on structured neonatal lung-disease queries, with the best-performing platforms scoring 5.75 to 5.78 out of 7 on expert neonatologist assessment [5]. These tools show useful accuracy on structured tasks; whether they are used safely in everyday practice is a separate and still-open question.

The honest answer is not always. LLMs can hallucinate, generating confident, fluent, factually wrong responses - a hallucinated dose, a fabricated guideline, or a plan that ignores a documented contraindication is indistinguishable from accurate output without active verification. Beyond LLMs, predictive ML models for sepsis, bronchopulmonary dysplasia (BPD), and mortality risk are entering neonatal intensive care units (NICUs) without always being validated in the populations they will serve; fewer than 20% of neonatal AI models have undergone external validation, and only one had reached bedside implementation readiness in a 2024 systematic review [6]. The challenge is therefore no longer whether AI will become part of neonatal practice - it already has - but how to ensure it is used safely, critically, and responsibly.

This report provides what neonatologists actually need: a practical interaction framework (GIRISH, which stands for Goal, Input, Role, Iterative refinement, Safety verification, and Human accountability), domain-specific prompt templates for immediate use, and the disciplinary context (NeonatAIlogy) to develop this responsibly. The objectives are threefold: (i) to provide a practical, tiered map of AI in neonatology; (ii) to introduce GIRISH, a bedside interaction framework with domain-specific prompt templates; and (iii) to propose NeonatAIlogy as a conceptual direction for responsible integration. This is a narrative, concept-focused technical report; it presents no new patient-level data and no statistical analysis, and GIRISH is offered as a first-generation proposal awaiting prospective validation.

## Technical report

Development approach

This is a narrative technical report and conceptual synthesis. It is neither a systematic review nor a PRISMA-ScR scoping review and does not claim exhaustive coverage. We therefore describe our approach transparently rather than through formal record-level accounting. We searched PubMed/MEDLINE, Embase, Scopus, and the Cochrane Library using Medical Subject Headings ("Artificial Intelligence," "Machine Learning," and "Neonatal Intensive Care Units") and free-text terms including "large language model" and "clinical AI governance." This search covered publications from January 2020 to January 2026 across eight neonatal clinical domains.

Sources were eligible if they were peer-reviewed studies, systematic reviews, randomized trials, or authoritative guidelines addressing AI or machine learning in one of eight predefined neonatal domains (respiratory, cardiovascular, neurological, gastrointestinal, infectious disease, metabolic, hematological, and nutrition/prematurity) or cross-cutting governance. Non-peer-reviewed commentaries and non-English records without an available translation were excluded. Included sources were synthesized narratively and thematically, with findings grouped by domain and AI tier. Recurring safety and governance themes were extracted to inform the development of the framework. Two pre-2020 foundational studies (the i-ROP deep-learning system [7,8] and the ANSeR randomized controlled trial (RCT) [9]) were included for clinical completeness. The GIRISH framework was developed through primary conceptual synthesis of these themes rather than being derived from a single prior source. As this is a narrative synthesis, a formal PRISMA-ScR flow diagram and record counts were not generated [10]; and we identify formal PRISMA-ScR mapping as a priority for future work.

This report did not involve human participants, and no external AI/ML engineers or additional domain experts were formally consulted during drafting. However, multidisciplinary expert input - including AI/ML engineers, clinical-informatics specialists, and ethicists - is built into the proposed modified-Delphi validation pathway described in the Validation Roadmap section.

Framework Availability

The GIRISH framework, its prompt templates, the three-tier safety hierarchy, the MV-GIRISH adaptation, and the quick-reference card are not patented and are subject to no proprietary or restrictive license. The authors grant permission for free reproduction, adaptation, and use for clinical, educational, and research purposes under the journal's Creative Commons Attribution (CC BY 4.0) license, with attribution given to the original article.

A practical map of AI in neonatology

Several systematic reviews have demonstrated expanding AI applications throughout neonatal intensive care [11]. Table 1, synthesized illustratively from published literature on AI applications in neonatology, maps five tiers of AI in neonatology and emphasizes what matters most in practice - specifically, what each tier means for a clinician working in an NICU today.

**Table 1:** Five tiers of AI in neonatology and their bedside relevance Performance ranges are drawn from the cited source studies and are illustrative. Before any local use, calibration metrics (e.g., Brier score) and external validation in a comparable population should be sought in addition to AUROC. AUROC: Area under the receiver operating characteristic curve; BPD: Bronchopulmonary dysplasia; CDSS: Clinical decision support system; CNN: Convolutional neural network; EEG: Electroencephalography; GIRISH: Goal, Input, Role, Iterative refinement, Safety verification, and Human accountability; HIE: Hypoxic-ischemic encephalopathy; LLM: Large language model; ML: Machine learning; MRI: Magnetic resonance imaging; RCT: Randomized controlled trial; RNN: Recurrent neural network; ROP: Retinopathy of prematurity.

AI tier	Examples	Neonatal application	Bedside relevance and current status
Tier 1: Rule-based CDSS	Protocol alerts; decision trees	Phototherapy thresholds; medication-dose alerts; ventilator alarm limits	Already built into monitors and pharmacy systems. Useful and validated – but it only knows the rules it was given.
Tier 2: Predictive ML	Random forest; XGBoost; logistic regression	Sepsis early-warning (AUROC 0.87–0.94); BPD risk (0.82–0.91); mortality scoring [12]	Being offered to NICUs now. Strong at risk ranking, but confirm it was validated in populations like yours; ask for calibration data, not just AUROC.
Tier 3: Deep learning	CNN; RNN; transformer models	ROP screening (>93% sensitivity) [7,8]; HIE MRI grading; EEG seizure detection (RCT-proven) [9]	The most mature AI in neonatology. i-ROP is externally validated across countries [7,8]; ANSeR improved seizure detection in an eight-center RCT [9]. These work with appropriate senior oversight.
Tier 4: Conversational AI/LLMs	ChatGPT; Gemini; Claude; Copilot; Perplexity; Consensus AI	Drug-dose queries; differentials; guideline summaries; parent-counseling drafts; discharge letters	The tier most commonly used informally at the bedside. High utility with a real risk of hallucination; the GIRISH framework is designed for this tier. Consensus AI searches peer-reviewed literature specifically and suits the evidence-retrieval role when a cited answer is required.
Tier 5: Agentic multimodal AI	Integrated vision-language and biosignal fusion	Real-time multiparameter monitoring; autonomous alert generation; agentic clinical support	Investigational and not yet validated for NICU deployment. Emerging governance frameworks should accompany any future clinical adoption.

Appraising the evidence: beyond AUROC

Published AI models for neonatology report impressive discrimination: sepsis prediction AUROC 0.87 to 0.94, BPD risk 0.82 to 0.91, and mortality prediction 0.85 to 0.92 [12]. AUROC measures risk ranking, not calibration; it cannot tell a clinician whether a predicted 80% risk truly reflects an 80% chance for a given patient, and calibration was reported in fewer than half of the reviewed studies [13]. Before adopting any predictive ML model, clinicians should ask for the Brier score and confirm external validation in a population similar to their own. Despite these strengths on paper, a 2024 systematic review found only one neonatal AI model at bedside implementation readiness, a reminder that impressive test-set accuracy and clinical deployability are not the same thing. A concurrent scoping review identified three persistent barriers: electronic health record non-interoperability, non-diverse training cohorts, and undefined regulatory pathways for adaptive AI [14]. AI is accelerating faster than governance can follow.

Capabilities and limitations of LLMs

LLMs are the AI tools that are most immediately relevant to daily practice and deserve a clear-eyed assessment. They are increasingly explored for clinical information retrieval and decision support, and recent systematic reviews highlight both the opportunities and the limitations of incorporating them into real-world clinical workflows [1]. A 2025 evaluation of eight platforms on neonatal lung disease and home oxygen therapy queries found the best performers (Bing Chat and Claude 3.5 Sonnet) scoring 5.78 and 5.75 out of 7 on expert neonatologist assessment, with significant variability across models [5]. These are genuinely useful scores, but they carry a critical caveat: a factually wrong response can appear just as confident on surface plausibility as a correct one.

LLMs genuinely help with several tasks: generating structured differentials for critique, drafting parent counseling in multiple languages, summarizing clinical evidence, writing discharge letters and referrals, teaching residents, and checking clinical reasoning against an alternative frame of reference. They also fall short in important ways. They cannot see the patient, access real-time monitors, or examine a fontanelle. They do not know the local antibiogram, the unit formulary, or the resuscitation-cot layout, and they may suggest a dose appropriate for a term infant but dangerous for a 26-week infant, attribute a guideline to a non-existent source, or underrepresent LMIC populations in their training data. These limitations - hallucination, opacity, and unclear accountability - together with unresolved medico-legal liability, mean that LLM output must be treated as advisory only and verified before any action [15]. The risk is not the technology but its use without structure - precisely what the GIRISH framework addresses.

Evidence from validated AI at the bedside

Predictive monitoring strategies for sepsis and necrotizing enterocolitis have shown promise in identifying deterioration before overt clinical manifestations appear [16]. Predictive ML models for late-onset sepsis integrate vital-sign trends, laboratory values, and clinical parameters into risk scores that may flag deterioration before conventional alert thresholds are met. AUROC values of 0.87 to 0.94 in validation cohorts [12,16] mean that a validated sepsis tool should rank the highest-risk infants correctly the large majority of the time. What such a tool cannot do is tell a clinician with certainty whether the alarm it has just raised is the right call for the baby in front of them. That judgment remains the clinician’s - informed, not replaced by the algorithm - and late-onset sepsis remains a major cause of morbidity among very preterm infants, providing a strong rationale for early predictive approaches.

In imaging and neurophysiology, AI has earned clinical trust. The i-ROP (retinopathy of prematurity) deep-learning system demonstrated sensitivity above 93% for plus disease, was non-inferior to expert graders, and has been validated multinationally [7,8]; this is AI at its most clinically mature, independently validated and accessible to NICUs without specialist graders. For hypoxic-ischemic encephalopathy (HIE), convolutional neural network (CNN)-based cranial MRI analysis shows emerging evidence of neuroradiologist-comparable performance in severity classification and 18- to 24-month neurodevelopmental-outcome prediction, with multicenter validation ongoing. Electroencephalography (EEG) seizure detection deserves special attention because it has been tested in a randomized trial rather than merely validated on a held-out dataset. The ANSeR multicenter RCT (n = 264, eight international centers) showed that an ML seizure-detection algorithm improved seizure-hour identification by 20.7% points over standard monitoring, with a per-neonate sensitivity of 81.3% and specificity of 84.4% [9]. Additional ML approaches have shown promising performance in predicting electrographic seizures [17]. AI can watch the EEG with consistent attention across a 12-hour overnight shift in a way no single clinician can, functioning here as a genuine clinical partner rather than merely an information tool.

The next wave is multimodal. In a 2025 Nature Medicine study, a transformer-based AI model was developed using a large multicenter dataset to generate individualized parenteral nutrition prescriptions for NICU infants. The system demonstrated close agreement with expert clinicians, performed well on external validation, and was associated with improved clinical decision-making, highlighting the potential of AI-assisted precision nutrition in neonatal care [18].

The GIRISH framework

Most clinicians using AI chatbots today either accept the first response at face value or feel uncertain and close the app; neither approach is safe or maximally useful because the former lacks verification and the latter lacks structure. Prompt engineering - the computational discipline of crafting inputs to optimize model performance - is not what most clinicians need. What they need is a clinical habit: a consistent six-step approach to every AI interaction that ensures the query is complete, anonymization is rigorous, output is interrogated, drug doses are verified, and a named clinician owns the decision. GIRISH provides that habit. Each letter represents a step taken before, during, and after every AI interaction at the bedside (Table 2).

**Table 2:** The GIRISH framework: six steps for safe, effective AI use at the NICU bedside BNFc: British National Formulary for Children; BPD: bronchopulmonary dysplasia; BW: Birth weight; CRP: C-reactive protein; DOB: Date of birth; DOL: Day of life; FiO_2_: Fraction of inspired oxygen; GA: Gestational age; GIRISH: Goal, Input, Role, Iterative refinement, Safety verification, and Human accountability; LLM: Large language model; MRN: Medical record number; PMA: Post-menstrual age; WBC: White blood cell count.

Step	What you do	Illustrative prompt	Clinical safety note
G – Goal	Define one bounded clinical task per interaction – a single, answerable question rather than “help me with this baby.”	“Generate a ranked differential for clinical deterioration in a 28-week infant, DOL 7, rising FiO_2_, CRP 48.”	One interaction, one goal. Broad, open-ended prompts return broad, unverifiable answers.
I – Input	Provide structured clinical context and anonymize rigorously before entering anything.	GA 28w, BW 950 g, DOL 7, FiO_2_ 0.55 (was 0.38), CRP 48, WBC 18.4, temp 37.9℃, on ampicillin day 7. No name, DOB, or MRN.	Never enter identifiers into any LLM. In small NICUs, GA, BW, and DOL together may re-identify a patient – state what is unavailable (e.g., “echo not yet done”) rather than omit it.
R – Role	Tell the AI what role it is playing and explicitly limit its scope.	“Act as a neonatal evidence-retrieval assistant. Generate differentials and suggest investigations. Do not prescribe. Flag uncertainty and an evidence grade for every suggestion.”	Explicitly limiting the role reduces hallucination risk. An AI instructed not to prescribe is less likely to volunteer an uncaveated drug dose.
I – Iterative refinement	Do not accept the first response; interrogate it, and follow up with specific challenges.	After the AI ranks late-onset sepsis first, ask: “Can BPD be diagnosed at DOL 7?” It corrects itself: not applicable until 36 weeks PMA. Then ask for the evidence grade for adding Gram-negative cover.	If output contradicts clinical gestalt, do not default to the AI. Ask it to cite its source and state what it is uncertain about. Disagreement is information.
S – Safety verification	Verify every drug dose, antibiotic, and threshold against your formulary and unit protocol before acting.	Verify gentamicin dose (GA-specific, Neofax/BNFc). Check the local antibiogram for Gram-negative cover. Review the record for contraindications.	Hallucination red flags: a dose outside the GA-expected range; a guideline attributed to a non-existent or outdated source; internal inconsistency (e.g., ibuprofen despite documented oliguria); overconfident probability language without an evidence grade.
H – Human accountability	Document that a named clinician made the decision. The AI assisted; the clinician decided.	Record: “AI-assisted differential generation, GIRISH framework, [date]. Platform: [name]. Output verified against [formulary/guideline]. Decision by [Name], [Designation], [Time].”	In multidisciplinary settings, designate a GIRISH interaction lead. The AI has no accountability; the clinician does. Documentation should follow local institutional and medico-legal policy: as consumer LLMs are not approved medical devices, units should record AI assistance in line with their own information-governance and AI-use policies rather than adopting fixed wording uncritically (see “Regulatory context”).

We acknowledge a legitimate concern that requiring manual verification limits workflow efficiency and could, if applied naively, introduce anchoring or automation bias - the tendency to over-rely on automated advice [19]. GIRISH is designed as a mitigation of this bias rather than a source of it. The systematic review that characterized automation bias identified explicit user accountability and structured verification as its most effective mitigators [19], which are precisely the iterative refinement, safety verification, and human accountability steps. GIRISH deliberately trades raw speed for verifiability and is intended for high-stakes, non-routine queries where an unverified answer is more dangerous than a slower one - not as a routine replacement for validated point-of-care references.

GIRISH applied: a worked clinical example

The following worked example is hypothetical and illustrative. The clinical parameters and AI responses are constructed to demonstrate the framework’s structure and do not represent a verbatim, reproducible transcript from any specific model, model version, or date. In practice, the interaction record should capture the platform, model name and version, and date (for example, “ChatGPT, GPT-4o, [date]”) to support reproducibility and audit. Table 3 walks through the framework using the deteriorating 28-week infant introduced above, showing how each step shapes a safe, accountable interaction.

**Table 3:** GIRISH applied to a deteriorating 28-week infant AAP: American Academy of Pediatrics; BP: Blood pressure; BPD: Bronchopulmonary dysplasia; BW: Birth weight; CRP: C-reactive protein; CXR: Chest radiograph; DOL: Day of life; FiO_2_: Fraction of inspired oxygen; GA: Gestational age; GIRISH: Goal, Input, Role, Iterative refinement, Safety verification, and Human accountability; HR: Heart rate; MAP: Mean arterial pressure; NNF: National Neonatology Forum (India); PDA: Patent ductus arteriosus; PMA: Post-menstrual age; RDS: Respiratory distress syndrome; SIMV: Synchronized intermittent mandatory ventilation; WBC: White blood cell count.

GIRISH Step	Application in this case
Scenario	28w, 950 g male, DOL 7, on SIMV, FiO_2_ 0.55 (up from 0.38 over 6 h). CRP 48 mg/L, WBC 18.4 x 10^9^/L (74% neutrophils), temp 37.9℃, feeds 80 mL/kg/day. Last CXR (DOL 5): moderate RDS. Medications: caffeine, ampicillin day 7 (gentamicin completed day 3, blood cultures negative).
G – Goal	“Generate a ranked differential for acute clinical deterioration and recommend evidence-based initial investigations. Do not suggest management yet.”
I – Input	GA 28w, BW 950 g, DOL 7. Ventilated (SIMV). FiO_2_ rising from 0.38 to 0.55 over 6 h. CRP 48, WBC 18.4 x 10^9^/L (74% neutrophils), temp 37.9℃, HR 176, BP 38/22 (MAP 27). Feeds 80 mL/kg/day, tolerating. Last CXR DOL 5: moderate RDS, no new changes. Ampicillin day 7, caffeine. Gentamicin completed day 3 (cultures negative). Echo not yet performed today. Fully anonymized.
R – Role	“Act as a neonatal evidence-retrieval assistant. Rank differentials by clinical probability. Flag an evidence grade for each. State what you are uncertain about.”
AI response (abbreviated)	(1) Late-onset sepsis: high probability given CRP, WBC, and rising FiO_2_, with Gram-negative organisms most likely after 7 days of ampicillin. (2) Ventilator-associated pneumonia: moderate; ventilated over 5 days, CXR unremarkable but cannot exclude. (3) Evolving chronic lung-disease trajectory: moderate; FiO_2_ trend consistent with evolving lung injury at 28w – note that a formal BPD diagnosis requires reassessment at 36 weeks PMA and is not applicable at DOL 7. (4) PDA hemodynamic compromise: lower probability without echo; cannot exclude.
I – Iterative refinement	The clinician asks for the evidence grade for adding Gram-negative cover after 7 days of ampicillin in a 28-weeker; the AI references AAP/NNF India guidance and prompts a local antibiogram check. Challenged on terminology (“Can BPD be diagnosed at DOL 7?”), the AI clarifies that BPD requires assessment at 36 weeks PMA or 28 days of age and that “evolving chronic lung disease” or “worsening RDS” is more accurate at DOL 7. The clinician then asks which findings would raise or lower the probability of PDA compromise without echo.
S – Safety verification	Gentamicin dose verified against Neofax for GA 28w. Local antibiogram checked for the Gram-negative sensitivity pattern. No hallucination red flags identified. Ibuprofen not suggested (correct, given the low MAP).
H – Human accountability	Record: “GIRISH AI-assisted differential generation, information-retrieval adjunct. Clinical decision to commence gentamicin in addition to ampicillin and to perform echo at 06:00 by (Consultant Name), Neonatology.”

The same discipline applies to communication tasks. For a father asking about the prognosis of a 24-week, 620 g infant on high-frequency oscillatory ventilation, the goal is bounded (“summarize published 24-week survivor outcomes with honest uncertainty ranges, in plain language; no individual prognosis”), and the role limits the AI to a plain-language information assistant that states outcome ranges rather than certainties. The evidence-retrieval platform returns EPICure2/NICHD (National Institute of Child Health and Human Development) ranges (approximately 50%-60% survival and 20%-30% disability-free survival at 24 weeks) [20-22]. These are population-level ranges offered as counseling context, not a prognosis for an individual infant. The safety step verifies these figures against EPICure2 [20,21], with no specific prognosis or inappropriate certainty language, and the record documents AI-assisted evidence retrieval with counseling delivered by the named consultant.

Domain-specific prompt templates

The templates in Table 4 are teaching aids for structuring queries, not authorization for AI-guided prescribing. Direct therapeutic decision-making is explicitly excluded from their scope - every template ends at differential generation, evidence retrieval, or information synthesis, and any subsequent drug, dose, or procedure must be verified against the formulary and confirmed by a senior clinician. Local institutional oversight and, where required, ethics committee or clinical governance approval should precede any structured use of these templates in patient care.

**Table 4:** Domain-specific GIRISH prompt templates for neonatal clinical practice AAP: American Academy of Pediatrics; aEEG: Amplitude-integrated electroencephalography; AUC: Area under the curve; AXR: Abdominal radiograph; BP: Blood pressure; BPD: Bronchopulmonary dysplasia; BW: Birth weight; CHD: Congenital heart disease; CMV: Cytomegalovirus; CRP: C-reactive protein; CVL: Central venous line; CXR: Chest radiograph; DHA: Docosahexaenoic acid; DIC: Disseminated intravascular coagulation; DOL: Day of life; EBM: Expressed breast milk; ELBW: Extremely low birth weight; EOS: Early-onset sepsis; ESPGHAN: European Society for Paediatric Gastroenterology Hepatology and Nutrition; ETTNO: Effects of transfusion thresholds on neurocognitive outcome trial; FiO_2_: Fraction of inspired oxygen; GA: Gestational age; GI: Gastrointestinal; GIR: Glucose infusion rate; GORD: Gastro-esophageal reflux disease; HFOV: High-frequency oscillatory ventilation; HIE: Hypoxic-ischemic encephalopathy; HR: Heart rate; IEM: Inborn errors of metabolism; IM: Intramuscular; iNO: Inhaled nitric oxide; IV: Intravenous; IVH: Intraventricular hemorrhage; LA:Ao: Left-atrium-to-aorta ratio; LMIC: Low- and middle-income country; LOS: Late-onset sepsis; MAP: Mean arterial pressure; MRI: Magnetic resonance imaging; NAS: Neonatal abstinence syndrome; NEC: Necrotizing enterocolitis; NICHD: National Institute of Child Health and Human Development; NNF: National Neonatology Forum (India); OI: Oxygenation index; pCO_2_: Partial pressure of carbon dioxide; PDA: Patent ductus arteriosus; PICC: Peripherally inserted central catheter; PMA: Post-menstrual age; PPHN: Persistent pulmonary hypertension of the newborn; RDS: Respiratory distress syndrome; SIP: Spontaneous intestinal perforation; TOBY: Total body hypothermia for neonatal encephalopathy trial; TOP: Transfusion of prematures trial; TORCH: Toxoplasmosis, other, rubella, cytomegalovirus, herpes simplex; TPN: Total parenteral nutrition; UAC: Umbilical arterial catheter.

Clinical domain	Goal statement (G) – copy and adapt	Key input parameters (I)	Safety verification (S) – always do this
Respiratory (RDS, BPD, PPHN, apnea)	“Summarize evidence for [surfactant/iNO/HFOV vs SIMV] in a [GA]-week infant with [diagnosis]. State evidence grade. Do not prescribe.”	GA, BW, DOL, FiO_2_, MAP, OI, blood gas (pH, pCO_2_, base excess), surfactant history, CXR result, ventilator mode and settings, caffeine dose	iNO: OI at or above 25, term/near-term (AAP 2019). Surfactant: weight/GA-specific dosing. HFOV: set MAP, amplitude, frequency. PPHN: exclude sepsis and metabolic causes first. Use WHO guidance for LMIC settings.
Cardiovascular (PDA, CHD, shock)	“Evidence for and against medical PDA closure vs expectant management in a [GA]-week infant, [DOL], with the echo findings below. Include evidence grade. Do not prescribe.”	Echo findings (diameter, flow, LA:Ao ratio), HR, BP (MAP), capillary refill, urine output, 24 h fluid balance, respiratory support, prior indomethacin/ibuprofen	Indomethacin: contraindicated if urine output below 1 mL/kg/h. Ibuprofen: contraindicated with active GI bleed. IV paracetamol: dose by GA. Ligation: per unit protocol. Verify with senior or cardiologist.
Neurological (HIE, IVH, seizures, NAS)	“Assess therapeutic-hypothermia eligibility (TOBY [23]/NICHD [24] criteria). List criteria met, not met, and uncertainties. State what additional information is needed.”	Mode of birth, time to first cry, Apgar scores (1, 5, 10 min), cord/first-gas pH and base excess, aEEG description, neurological examination, age in hours, GA (must be at or above 36w)	Cooling window: start within 6 h of age. Eligibility: pH at or below 7.00 or base deficit at or above 16 mmol/L, and/or abnormal neurology and/or abnormal aEEG. Phenobarbitone loading: dose by weight, not GA. Levetiracetam: evidence grade B only. MRI: days 3 to 5 optimal.
Gastrointestinal (NEC, SIP, GORD, jaundice)	“Stage this infant using modified Bell’s criteria. Recommend initial management. State surgical-referral triggers. Flag evidence grade throughout.”	Abdominal findings (distension, tenderness, erythema, palpable loops), AXR result (pneumatosis, portal gas, free air), WBC, CRP, platelet count and trend, feed history and volume at presentation, stool pattern	Triple antibiotics: ampicillin, gentamicin, and metronidazole (confirm local protocol). Nil by mouth: minimum 7 to 10 days for Bell IIa/IIb. Bell IIb/III: urgent surgical consult. Antifungal prophylaxis per unit protocol in ELBW/less-than-28-week infants in high-prevalence units. Peritoneal drain vs laparotomy: discuss with surgery per local protocol.
Infectious disease (EOS, LOS, fungal, TORCH)	“Recommend empirical antibiotics for suspected LOS in the infant below. Include evidence grade and duration. State what requires culture results to determine.”	GA, PMA, DOL, prior antibiotics (agent, duration), culture results, CRP trend (last 3), access device (UAC/PICC/CVL), local antibiogram, signs of a specific focus	Gentamicin: extended-interval dosing by GA (Neofax). Vancomycin: AUC-guided monitoring preferred over trough. Amphotericin B: check renal function first. De-escalate on sensitivities within 48 to 72 hours – prompt the AI to state this.
Metabolic (hypoglycemia, IEM, TPN, electrolytes)	“Stepwise investigation algorithm for persistent hypoglycemia unresponsive to GIR above 8 mg/kg/min. Flag diagnoses requiring urgent exclusion.”	Blood-glucose trend (last 6 readings with GIR at each), current GIR, insulin level if measured, cortisol, growth hormone, lactate, ammonia, urine ketones, amino-acid screen, family history, maternal medications, gestational complications	Glucagon 200 mcg/kg IV/IM; blunted response in preterm. Diazoxide: monitor for PPHN. Octreotide: case-series only – specialist input. IEM screen: critical sample during hypoglycemia. Metabolic referral if undiagnosed at 48 hours.
Hematologic (anemia, DIC, polycythemia)	“Summarize transfusion-threshold evidence for a [GA/PMA]-week infant on [ventilatory support]. Reference TOP [25] and ETTNO [26] trial results.”	Hemoglobin trend (last 3), respiratory support and FiO_2_, 24 h apnea frequency, weight trend, reticulocyte count, blood group and Coombs, bilirubin, erythropoietin status	TOP [25] and ETTNO [26] (2020–2022): liberal and restrictive thresholds showed equivalent neurodevelopmental outcomes, with fewer transfusions under the restrictive approach. Use CMV-negative/irradiated products for immunocompromised infants. Platelet threshold: 25 x10^9^/L if stable; 50 x10^9^/L pre-procedure; 100 x10^9^/L with active bleeding.
Nutrition and prematurity (TPN, enteral feeding, growth)	“Evidence-based enteral-advancement protocol for a [GA]-week infant, [DOL], with the feeding history below. State feed-intolerance triggers.”	GA, BW, DOL, feed volume (mL/kg/day), feed type (EBM/donor/formula), tolerance indicators, 7-day weight trend, TPN composition, NEC risk factors, fortification status	Advancement rate: up to 30 mL/kg/day increase in the very preterm. Fortification: commence at 100 mL/kg/day. Vitamin D: 400 to 800 IU/day. DHA: evidence exists, but quality is variable. Probiotics: check the national-body position (NNF India, ESPGHAN).

Table 4, synthesized from published clinical trials and current guidelines, provides ready-to-adapt prompt templates for the eight major neonatal clinical domains. Each entry gives a structured goal statement, the key parameters to include in the input, and the safety verification steps. These are starting points to be modified for a specific patient; the safety-verification steps are mandatory. In general, consensus AI suits goals that require peer-reviewed evidence synthesis, whereas general LLMs (ChatGPT, Claude, and Gemini) suit goals that require reasoning, differential generation, or drafting. For the safety step, always cross-reference Neofax, BNFc, or the unit formulary because no LLM is a primary dose reference.

Five habits make every prompt work better. First, be specific about gestational age (GA) and DOL in every query; if the response for a 24-weeker and a 34-weeker looks the same, the prompt was too vague. Second, tell the AI what it cannot do - for example, “do not prescribe; rank differentials only” - because limiting scope reduces the range of possible hallucination. Third, ask for an evidence grade for each suggestion, and treat output that the model cannot grade with skepticism. Fourth, state explicitly what is unavailable, since omitting "echo not done" or "antibiogram unavailable" invites the model to fill the gap incorrectly. Fifth, use follow-up prompts deliberately, treating the first response as a draft and challenging it with questions such as “what am I missing?” or “what would change your top differential?”

Safety verification: a three-tier hierarchy

Three mandatory tiers govern action on any AI output. The first treats every output as advisory only, never authoritative. The second cross-references the output against current guidelines (WHO, AAP, BAPM (British Association of Perinatal Medicine), NNF (National Neonatology Forum) India, or equivalent). The third requires a senior clinician to confirm before any drug, procedure, or escalation. This hierarchy is non-negotiable: no output bypasses the third tier.

GIRISH for resource-limited settings: MV-GIRISH

Recommending higher-risk consumer tools to under-resourced units while better-resourced centers use enterprise-grade platforms would establish an unacceptable double standard of care, and we do not endorse that. MV-GIRISH is therefore not a recommendation to substitute public LLMs for validated care, but a harm-reduction stopgap for settings where no enterprise-grade option yet exists. Its non-negotiable conditions are: (i) no patient-identifiable information is ever entered; (ii) no clinical detail is entered into any platform that does not meet an applicable data-protection standard; (iii) AI output is used only for general educational framing and never as the primary basis for a drug, dose, or acute-management decision; and (iv) the safety hierarchy and human-accountability steps remain fully in force. The input step uses whatever non-identifiable parameters are available - GA, birth weight (BW), DOL, examination findings, temperature, glucose, and full blood count or CRP where possible - while explicitly declaring which tests are unavailable, and the interaction is documented as "GIRISH-adapted (resource-limited)." The second safety tier defaults to WHO guidance and the relevant national body, such as the NNF India, the Perinatal Society of Southern Africa, or the Nigerian Society of Neonatology. We frame the goal as equitable access to safe, validated AI tools - not access to less-safe tools - and call for open-source and low-bandwidth, enterprise-grade options for LMIC NICUs so that this stopgap becomes unnecessary. The adaptation of digital clinical tools for low-resource settings has previously been explored using consensus-based approaches [27].

Regulatory context and where GIRISH sits

GIRISH is a human-governance overlay for clinician-initiated AI use, not software marketed for a medical purpose; it is therefore distinct from Software as a Medical Device (SaMD). This distinction matters because the consumer LLMs most clinicians actually use at the bedside are not cleared or CE-marked medical devices and fall outside current device-approval pathways. Under the US FDA’s AI/ML-based SaMD framework and Action Plan [28], and the EU Artificial Intelligence Act (Regulation (EU) 2024/1689), which classifies AI intended to inform clinical decisions as high-risk and requires risk management, data governance, transparency, and human oversight [29], any AI tool formally deployed for a clinical purpose must meet the corresponding obligations. GIRISH does not confer regulatory compliance and should not be read as doing so; rather, it operationalizes the human-oversight, transparency, and accountability principles that these frameworks demand and is intended to complement - never substitute for - institutional SaMD governance, information-governance sign-off, and, where applicable, regulatory clearance.

Validation roadmap

GIRISH is presented as a first-generation clinical framework; the worked example illustrates structure, not proven efficacy. GRADE (Grading of Recommendations Assessment, Development, and Evaluation), SBAR (Situation, Background, Assessment, and Recommendation), and neonatal sepsis bundles each preceded the validation that later established them. We propose a three-stage validation pathway. First, a modified Delphi process involving at least 20 global experts - including neonatologists, clinical-informatics specialists, ethicists, and AI/ML engineers - would establish face and content validity using two to three anonymous rating rounds on a nine-point Likert scale with a pre-specified consensus threshold, item-level and scale-level content validity indices calculated for each GIRISH step and each domain template, and thematic analysis of free-text expert commentary to identify items requiring rewording, merging, or removal. Second, simulation-based usability testing with think-aloud protocols and structured debriefing would assess comprehension, workflow burden, and hallucination-detection performance. Third, prospective implementation across at least two NICU centers would evaluate documentation quality, safety events, near-miss reports, and clinician acceptability as primary outcomes, with inter-rater agreement on framework adherence as a process measure. We invite international collaboration on this pathway. Pending completion of the first two stages, GIRISH should not be adopted as unit policy or as routine clinical practice. Until independent expert validation is established, its use should be confined to clinician education and training, structured evaluation under local clinical-governance oversight, and the high-stakes non-routine queries for which the framework was designed, with the safety hierarchy and human-accountability requirements applying in full.

Quick-reference summary

Table 5 condenses GIRISH into a single card suitable for a NICU workstation; it may be freely copied for clinical use.

**Table 5:** GIRISH quick-reference summary GA: Gestational age; GIRISH: Goal, Input, Role, Iterative refinement, Safety verification, and Human accountability; LLM: Large language model; MRN: Medical record number.

Step	What you do	Non-negotiable safety check
G – Goal	One bounded task per interaction – one specific, answerable question.	Vague goals return unverifiable answers.
I – Input	Structured clinical context, fully anonymized. State what is unavailable.	Never enter name, date of birth, or MRN into any LLM.
R – Role	Tell the AI its role and limits (“do not prescribe; flag uncertainty and evidence grade”).	Explicitly limiting scope reduces hallucination risk.
I – Iterate	Interrogate the output. Challenge it. Ask what it is uncertain about.	If the AI contradicts your gestalt, ask it to cite its source. Disagreement is information.
S – Safety	Every drug dose and guideline – verify against your formulary before acting.	Red flags: dose outside the GA range; a non-existent source; internal inconsistency; overconfident language.
H – Human	A named clinician made the decision. Document it.	“AI-assisted [task], GIRISH, [date]. Verified against [source]. Decision by [Name], [Designation].” The AI has no accountability; you do.

## Discussion

NeonatAIlogy: building the discipline that supports this practice

If GIRISH provides the bedside framework, NeonatAIlogy is proposed to help the specialty develop AI capability responsibly and equitably. We define NeonatAIlogy as the systematic study, integration, governance, and teaching of AI in neonatal and perinatal medicine, with patient safety, accountability, and equity as its primary principles. We advance this as a proposed organizing concept and direction of travel, not as an already established subdiscipline. The concept aligns with the broader vision of high-performance medicine, in which AI augments rather than replaces clinician expertise [30]. What would distinguish it from "AI in the NICU" is disciplinary coherence: a defined evidence base, a methodological toolkit, and institutional governance. Other focused areas of neonatology have matured from informal practice into recognized sub-areas over time; we draw this only as a loose analogy, not as evidence, and make no claim that NeonatAIlogy has achieved comparable status. Any move from concept toward formal recognition would require regulatory alignment, institutional adoption, and a validated evidence base, none of which this report claims to provide. This report therefore offers a practical approach and a conceptual framework for NeonatAIlogy, intended to structure subsequent validation rather than to pre-empt it.

A NeonatAIlogy curriculum would cover AI taxonomy, GIRISH application, model-evidence appraisal, ethics, and hallucination-detection simulation. Institutional governance would require an AI clinical safety board, a NeonatAI registry, and mandatory near-miss reporting, while the research agenda would prioritize multicenter validation, randomized trials of AI-assisted decision-making, and equity analyses across gestational-age strata and across LMIC and high-income settings. An AI near-miss log recording platform, task, error type, and outcome would generate prospective safety evidence that no controlled trial can produce at this pace of development, and we propose it as an expected component of any structured evaluation of GIRISH.

Ethical considerations

Three ethical points deserve emphasis. First, the AI a unit adopts may not work for its patients. Models trained on high-income NICU data degrade in LMIC or ethnically diverse populations, and a model validated in one setting may perform substantially worse in another, so local validation is mandatory before any AI tool enters clinical use. ML-based clinical decision support has shown substantial promise but requires population-specific validation before widespread adoption [31].

Second, clinicians should be able to understand why an AI system produced the output it did. Explainable AI methods generate clinician-interpretable rationales for deep-learning outputs, and the iterative refinement step of GIRISH builds this interrogation into routine practice. Parental consent principles for AI-assisted clinical care are articulated in WHO guidance [32] and, increasingly, in national frameworks - for example, those of the United Kingdom's National Health Service (NHS) and the Australian National Health and Medical Research Council (NHMRC) - which institutions should adapt to local requirements. Only enterprise-grade LLM platforms that meet national data-protection standards, such as the Health Insurance Portability and Accountability Act of 1996 (HIPAA), the General Data Protection Regulation (GDPR), or an equivalent, should be used for any input containing clinical detail.

Third, responsible adoption requires more than a framework. Before GIRISH is used at the bedside, units should secure appropriate parental consent or information-sharing arrangements for AI-assisted care, consistent with local law and WHO guidance [32]; obtain information-governance and data-protection sign-off before any clinical detail is entered into an AI tool; and provide structured clinician training and competency assessment (see the proposed NeonatAIlogy curriculum). Adequate digital infrastructure, an approved enterprise platform, and a named clinical-governance owner are minimum practical prerequisites. Investigators applying or validating GIRISH should additionally follow established reporting and ethics guidance for AI in healthcare research, alongside WHO guidance [15,32].

Finally, the algorithm will never be accountable - the clinician will. The GIRISH human-accountability step is not bureaucratic documentation but the boundary that keeps neonatal medicine human. The relationship between neonatologist and family - the compassionate presence, the experiential judgement, and the willingness to sit with uncertainty - cannot be replicated by any AI. The clinician who uses GIRISH brings AI into the service of that relationship, governed and accountable at every step, and this is the principle NeonatAIlogy must protect.

Challenges and mitigations

Table 6 summarizes the principal barriers to safe AI integration in neonatology alongside proposed mitigations spanning data infrastructure, algorithmic quality, regulation, clinician adoption, LLM-specific risks, and equity.

**Table 6:** Barriers to safe AI integration in neonatology and proposed mitigations AUROC: Area under the receiver operating characteristic curve; CE: Conformité Européenne; EHR: Electronic health record; FDA: US Food and Drug Administration; FHIR: Fast Healthcare Interoperability Resources; GIRISH: Goal, Input, Role, Iterative refinement, Safety verification, and Human accountability; HL7: Health Level Seven; IMDRF: International Medical Device Regulators Forum; LLM: Large language model; LMIC: Low- and middle-income country.

Domain	Specific barrier	Proposed mitigation
Data infrastructure	EHR non-interoperability; data silos; LMIC underrepresentation in training data	HL7 FHIR standards; Vermont Oxford Network data sharing; federated learning; globally representative collection mandates
Algorithmic quality	Class imbalance; unreported calibration; poor generalizability to the local population	Multicenter consortia; external-validation mandates; standardized reporting (Brier score with AUROC); post-deployment monitoring
Regulatory	Undefined pathways for adaptive AI; approval slow relative to model improvement	FDA/CE adaptive frameworks; pre- and post-market reporting; IMDRF harmonization; explicit mapping of clinical AI use to the FDA AI/ML SaMD Action Plan [28] and EU AI Act high-risk requirements [29]
Clinician adoption	Limited AI literacy; alert fatigue; no AI training in fellowship curricula; liability concerns	NeonatAIlogy curriculum; GIRISH as an accessible entry point; co-design with bedside clinicians; transparent performance dashboards
LLM-specific risks	Hallucination; no real-time integration; confidentiality risk with unstructured prompts	Mandatory GIRISH deployment; red-flag training (Table *2*, S step); enterprise-only platforms; MV-GIRISH for resource-limited settings; accountability- and verification-based mitigation of automation bias [19]
Equity and access	Performance disparity across populations; enterprise-LLM cost in LMICs	LMIC-representative training; open-source development; low-bandwidth deployment; MV-GIRISH

Limitations

This is a narrative, non-systematic technical report and is susceptible to selection and publication bias. It does not follow PRISMA-ScR or report formal record counts, and its map of the field should not be read as exhaustive [10]. GIRISH has not been prospectively validated, the domain templates lack independent expert consensus, and the worked examples are hypothetical illustrations of structure rather than evidence of efficacy or safety. The cited performance metrics are reproduced from source studies and are not independently verified here; many were reported without calibration data or confidence intervals, which limits their interpretation. The framework confers no regulatory compliance, and its documentation recommendations must be adapted to local medico-legal and SaMD policies. Governance principles are stated in a model-version-agnostic way, and future validation must actively include sub-Saharan Africa, South-East Asia, and Latin America.

## Conclusions

AI is already at the neonatal bedside - in the monitors, in the chatbot, and in the agentic systems now arriving in NICUs. The question is not whether to use it but how. GIRISH offers a six-step answer: a bounded goal, anonymized input, a limited role, interrogated output, verified doses, and a named decision-maker. Two anchors run through the framework. First, AUROC alone cannot judge a clinical AI model, so calibration data and external validation in a comparable population should be sought before adoption. Second, hallucination red flags - a dose outside the gestational-age range, a guideline attributed to a non-existent source, internal inconsistency with the patient's parameters, and overconfident language without an evidence grade - should trigger skepticism before any output is acted upon. NeonatAIlogy is proposed to organize the curricula, governance, and research this requires, so that safe AI reaches NICUs in Dehradun, Lagos, and Christchurch, not only those with enterprise budgets. GIRISH itself awaits validation, but the discipline it calls for - rigor, accountability, and humanity - is what neonatal care has always demanded.
